# Mesenteric Panniculitis in a Patient with Ulcerative Colitis in Remission on Vedolizumab Therapy: A Case Report and Literature Review

**DOI:** 10.3390/jcm15145511

**Published:** 2026-07-14

**Authors:** Carmen Atodiresei, Alina-Ecaterina Jucan, Georgiana Elena Sârbu, Claudiu Vasile Mihai, Ioana Ruxandra Mihai, Bogdan-Victor Ștefănescu, Mihaela Dranga, Otilia Nedelciuc, Georgiana Emmanuela Gîlcă-Blanariu, Alin Constantin Pînzariu, Cristina Cijevschi Prelipcean, Cătălina Mihai

**Affiliations:** 1 “Grigore T. Popa” University of Medicine and Pharmacy, 700115 Iași, Romania; carmen_atodiresei@yahoo.com (C.A.); ghiata.alina-ecaterina@d.umfiasi.ro (A.-E.J.); georgiana-elena.sarbu@umfiasi.ro (G.E.S.); mihai_vasile-claudiu@d.umfiasi.ro (C.V.M.); ioana-ruxandra_mihai@umfiasi.ro (I.R.M.); bogdan-victor.stefanescu@d.umfiasi.ro (B.-V.Ș.); mihaela_dra@yahoo.com (M.D.); otilianedelciuc@yahoo.com (O.N.); alin.pinzariu@umfiasi.ro (A.C.P.); cristina.cijevschi.prelipcean@umfiasi.ro (C.C.P.); catalina.mihai@umfiasi.ro (C.M.); 2 Institute of Gastroenterology and Hepatology, Sf. Spiridon County Emergency Clinical Hospital, 700111 Iași, Romania; 3 Department of Radiology, Sf. Spiridon County Emergency Clinical Hospital, 700111 Iași, Romania; 4 Department of Rheumatology and Rehabilitation, Clinical Rehabilitation Hospital, 700661 Iași, Romania; 5 “Socola” Institute of Psychiatry, 700282 Iași, Romania; 6 IIIrd Surgical Unit, Sf. Spiridon County Emergency Clinical Hospital, 700111 Iași, Romania

**Keywords:** mesenteric panniculitis, ulcerative colitis, Crohn’s disease, vedolizumab, IBD, sclerosing mesenteritis

## Abstract

**Background**: Mesenteric panniculitis (MP) is a chronic fibroinflammatory disorder of the mesenteric adipose tissue and is frequently considered an idiopathic condition. Its association with inflammatory bowel disease (IBD), particularly ulcerative colitis (UC), remains poorly characterized, with only limited evidence available in the literature. In addition to presenting a clinical case, we performed a narrative review of the literature regarding the relationship between MP and IBD, including epidemiology, pathophysiological mechanisms, diagnostic challenges, and therapeutic approaches. **Case Presentation**: We report the case of a 32-year-old woman with UC in deep clinical, endoscopic, and histological remission while receiving vedolizumab therapy, who developed symptomatic MP diagnosed by contrast-enhanced computed tomography. Infectious, neoplastic, and selected fibroinflammatory causes were excluded during the diagnostic work-up. Histological confirmation was not obtained. The patient was treated with prednisone and tamoxifen, resulting in complete clinical and radiological remission while vedolizumab therapy was continued. **Conclusions**: This case describes the rare co-occurrence of MP and UC in deep remission during ongoing vedolizumab treatment. Given the absence of histological confirmation and the frequently idiopathic nature of MP, a causal relationship with either UC activity or vedolizumab therapy cannot be established. The observation should therefore be regarded as hypothesis-generating. Further studies are required to clarify the potential relationship between MP, IBD, and biologic therapies.

## 1. Introduction

Inflammatory bowel diseases (IBD), primarily represented by Crohn’s disease (CD) and ulcerative colitis (UC), are chronic, systemic conditions with multifactorial etiology, characterized by complex interactions between genetic predisposition, intestinal dysbiosis, environmental factors, and dysregulated immune responses. The incidence and prevalence of IBD are continuously increasing worldwide, reaching approximately 0.8–1% in many developed countries, with an upward trend also observed in developing regions, resulting in a significant burden both at the individual level and on public healthcare systems [1,2,3].

IBD is characterized by recurrent inflammation of the gastrointestinal tract and is frequently associated with extraintestinal manifestations (EIMs), reflecting its systemic nature. EIMs are complications affecting organs and systems outside the gastrointestinal tract, including musculoskeletal, cutaneous, ocular, hepatobiliary, and cardiovascular structures, contributing significantly to the overall disease burden [4,5]. They may precede the onset of digestive symptoms, evolve concurrently with intestinal disease activity, or persist independently of it, including during periods of remission, thereby underscoring the systemic nature of inflammatory bowel diseases [4,5].

According to the 2024 ECCO Consensus, EIMs are classified based on their pathogenic mechanisms and relationship with disease activity into classical manifestations, complications of chronic systemic inflammation, associated manifestations, and treatment-induced manifestations, highlighting their heterogeneity and complexity. Within this framework, the modern approach transcends a strictly organ-specific perspective, integrating systemic manifestations into the disease spectrum. This approach advocates multidisciplinary evaluation and the use of standardized diagnostic and monitoring criteria [6].

Mesenteric panniculitis (MP) is a chronic fibroinflammatory condition of the mesenteric adipose tissue, characterized by non-specific inflammation, adipocyte necrosis, and variable degrees of fibrosis. It is classified within the spectrum of sclerosing mesenteritis, which reflects the relative proportion of these histopathological components. Its clinical, imaging, and histological expression is heterogeneous, leading to difficulties in definition and diagnostic standardization. It is increasingly interpreted as an immune-mediated process of the mesentery [7,8,9].

MP has been reported in association with a wide range of conditions, including chronic inflammatory diseases, autoimmune disorders, infections, and neoplasms, without identification of a single unifying etiology, supporting its multifactorial and potentially reactive nature [7,8,9].

Interest in the association between MP and IBD stems from the recognition of the mesentery as an active participant in systemic inflammatory processes. In this context, MP is interpreted as an expression of a chronic fibroinflammatory mesenteric response, possibly correlated with persistent intestinal inflammation, suggesting a potential relationship with IBD. Nevertheless, available data remain limited and heterogeneous, precluding the establishment of a clear causal relationship, primarily due to the lack of standardization of diagnostic criteria and incomplete reporting of patients’ clinical characteristics [7,10,11].

In parallel, the possible co-occurrence of MP in patients receiving biological therapies for IBD, including gut-selective agents, has not been systematically investigated and remains poorly documented in the literature.

In this context, MP emerges as an entity at the intersection of imaging, gastroenterology, and immunopathology, with its clinical significance and position within the IBD framework incompletely defined.

Against this background, the present report aims to describe a rare case of MP occurring in a patient with UC in deep remission during vedolizumab therapy, and to provide a critical narrative review of the available evidence on the association between MP and IBD. The rationale for this report stems from the paucity of data specifically linking MP to UC, the limited published evidence in the context of gut-selective biologic therapy, and the unresolved question of whether MP may represent a mesenteric fibroinflammatory phenomenon related to IBD, independent of mucosal disease activity. Given the frequently idiopathic nature of MP and the absence of histopathological confirmation in the present case, no causal relationship between MP, UC, and vedolizumab therapy can be established. Accordingly, the observations presented herein should be regarded as hypothesis-generating and warrant further dedicated investigation.

## 2. Case Presentation

We report the case of a 32-year-old female patient with normal body weight and a history of UC diagnosed at age 27, with pancolonic involvement (E3) and a corticosteroid-dependent disease course. Biological therapy with infliximab was initiated, but secondary loss of response occurred, and the patient was later switched to vedolizumab three years prior to the current presentation. The course of IBD under biological therapy was favorable, with clinical, endoscopic, and histological remission.

In July 2025, approximately four weeks after the last scheduled vedolizumab infusion (administered at the standard maintenance interval of eight weeks), the patient presented to the emergency department with diffuse crampy abdominal pain, associated with nausea, vomiting, and heartburn. On physical examination, the abdomen was moderately distended, with diminished bowel sounds and diffuse tenderness on both superficial and deep palpation, with no signs of peritoneal irritation. The remainder of the physical examination was within normal limits.

The principal laboratory, immunological, endoscopic, and histological findings at the time of presentation are summarized in Table 1.

**Figure 1:**
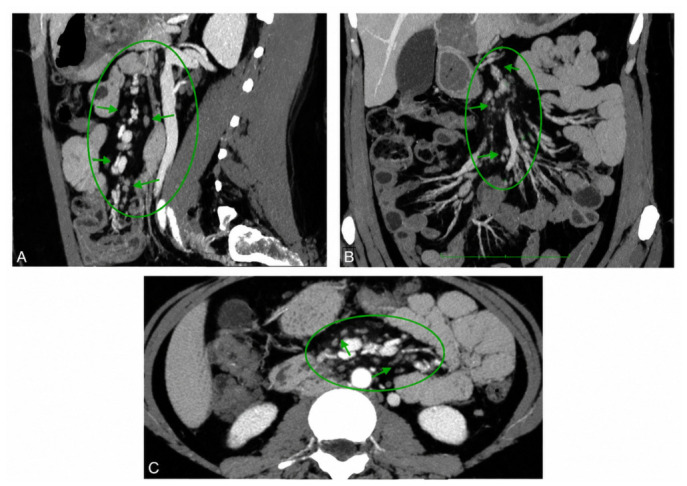
Contrast-enhanced computed tomography findings of mesenteric panniculitis. (**A**) Sagittal CT image demonstrating multiple sub-centimeter lymph nodes (green arrows) at the level of the mesenteric root. (**B**) Coronal CT image showing densification of the mesenteric fat with encasement of mesenteric vessels without direct invasion (green oval), and sub-centimeter lymphadenopathies (green arrows). (**C**) Axial CT image revealing diffuse hyperattenuating mesenteric fat infiltration (green oval) and associated lymph nodes (green arrows), suggestive of mesenteric panniculitis.

Contrast-enhanced abdominal and pelvic computed tomography (CT) revealed diffuse infiltration of the mesenteric root adipose tissue, extending into the retroperitoneal vascular compartment, associated with sub-centimeter lymphadenopathy and a pseudotumoral appearance, findings suggestive of MP. The imaging features are illustrated in Figure 1.

Tissue biopsy was not performed, as the patient elected to defer the procedure at initial presentation and opted for medical treatment. Accordingly, treatment was initiated with prednisone (40 mg/day, followed by a gradual dose reduction of 5 mg/week after the first four weeks) in combination with tamoxifen (10 mg twice daily for 12 weeks), with concomitant continuation of vedolizumab therapy.

This regimen was selected based on the available evidence supporting corticosteroid–tamoxifen combination therapy in symptomatic MP [12,13].

**Figure 2:**
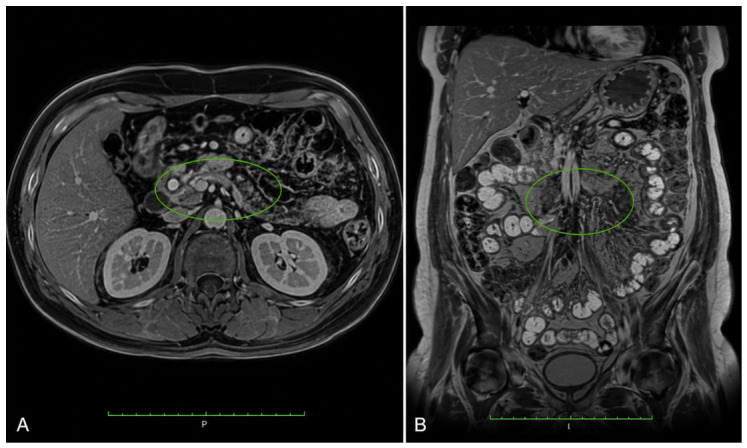
Mesenteric panniculitis—follow-up MRI at one month. (**A**) Axial opposed-phase T1-weighted image demonstrating complete resolution of the previously observed mesenteric fat infiltration, with no residual inflammatory lymph nodes at the mesenteric root (green oval). (**B**) Coronal T2-weighted fat-saturated image confirming complete resolution of the mesenteric fat infiltration and absence of residual inflammatory lymph nodes (green oval), consistent with complete radiological remission.

The clinical course was favorable, with complete resolution of gastrointestinal symptoms and normalization of inflammatory markers. Follow-up magnetic resonance imaging (MRI) performed at one month demonstrated complete resolution of mesenteric inflammatory changes, confirming clinical and radiological remission (Figure 2).

The distinctive feature of this case lies in the occurrence of MP in a patient with UC in clinical, endoscopic, and histological remission on vedolizumab therapy. To the best of our knowledge, no previous report has described this specific clinical scenario.

Whether MP represents a coincidental idiopathic process, a mesenteric fibroinflammatory phenomenon related to the systemic immune dysregulation associated with IBD, or a temporally associated event unrelated to the underlying disease remains an open question that cannot be resolved on the basis of a single case observation.

## 3. Mesenteric Panniculitis—General Overview

### 3.1. Definition and Epidemiology

MP is a chronic benign condition of the mesenteric adipose tissue, characterized histopathologically by the variable combination of non-specific inflammation, adipocyte necrosis, and fibrosis, with predominant involvement of the small bowel mesenteric root. It is currently classified within the spectrum of sclerosing mesenteritis, which reflects a pathological continuum determined by the relative proportion of these components, rather than the existence of distinct entities [11,14,15].

From a historical perspective, this condition was first described in 1924 by Jura as retractile sclerosing mesenteritis. Subsequent clinicopathological studies progressively unified the various terms used to describe this entity, leading to the current concept of sclerosing mesenteritis as a pathological continuum encompassing different manifestations of the same disease process [16,17,18,19].

The development and widespread use of abdominal CT have contributed significantly to the increasing recognition of this condition, suggesting that MP is likely underdiagnosed in routine clinical practice and more prevalent than previously considered [7,20].

The reported prevalence varies widely, ranging from 0.16% to 7.83%, with differences attributed to the heterogeneity of imaging criteria and methodological variability across studies [11,13,21,22,23].

CT-based studies reported prevalence rates of approximately 0.6% in the initial series, while the adoption of more sensitive radiological criteria led to higher estimates, reaching up to 7.83%, highlighting the impact of the diagnostic definition on reported frequency [7,19,23].

Large-scale retrospective analyses report intermediate prevalence rates, ranging from 1.1% to 2.5%, supporting the notion that this entity is more prevalent than previously recognized [20,24,25].

From a demographic standpoint, the condition predominantly affects middle-aged and elderly adults, with a peak incidence between 50 and 70 years of age, and is rarely described in the pediatric population [7,14].

Most studies report a moderate male predominance of approximately 2:1, although this finding is not consistent across cohorts [20,26,27].

Overall, the characteristic epidemiological profile is that of an incidental diagnosis in adult patients, in the context of the widespread use of abdominal CT, while the true frequency of the condition remains difficult to estimate precisely due to the methodological heterogeneity of available studies [22,24,28,29].

### 3.2. Etiopathogenesis

The etiopathogenesis of mesenteric panniculitis remains incompletely elucidated and is currently considered the expression of an idiopathic, probably immune-mediated, fibroinflammatory process of the mesenteric adipose tissue. In the absence of a single etiological determinant, multiple potential factors have been implicated. These include abdominal trauma, surgical interventions, mesenteric ischemia, chronic infections, autoimmune diseases, neoplastic processes, and drug reactions. Direct causal relationships have not been demonstrated; however, supporting the multifactorial and heterogeneous nature of the disease [19,30,31].

The central pathogenic mechanism involves immunometabolic dysfunction of the mesenteric adipose tissue, which acts as an active immunological organ. Resident adipocytes and macrophages secrete pro-inflammatory cytokines and adipokines, including TNF-α, IL-6, and IL-8, promoting cellular recruitment, low-grade chronic inflammation, and fibrotic remodeling. The transformation of macrophages into foam cells and the activation of PPAR-γ-dependent pathways suggest mechanisms shared with visceral adipose tissue inflammation and atherosclerotic processes [32,33,34].

Histopathologically, MP is characterized by chronic inflammation, adipocyte necrosis, and progressive fibrosis in variable proportions, justifying its classification within the sclerosing mesenteritis spectrum [14,15,34].

The immunometabolic model is supported by clinical associations with metabolic syndrome, visceral obesity, and metabolic dysfunction-associated steatotic liver disease (MASLD, formerly termed non-alcoholic fatty liver disease [NAFLD]), suggesting that systemic low-grade inflammation may promote mesenteric compartment activation. Additionally, associations with chronic infections, systemic fibroinflammatory diseases, and secondary postoperative or iatrogenic forms have been reported, including in the context of modern therapies, indicating the existence of a common inflammatory response to various triggering factors [8,14,32,35,36,37,38].

In the context of IBD, the mesentery is recognized as an active immunological compartment involved in the perpetuation of inflammation. Mesenteric hypertrophy, fibroadipose proliferation, and the secretion of pro-inflammatory mediators support the existence of an intestino-mesenteric continuum, in which inflammation extends beyond the intestinal mucosa to involve adjacent mesenteric structures [39,40,41,42].

Overall, MP may be interpreted as the result of the interaction between chronic immune activation, immunometabolic dysfunction, and local triggering factors.

In at least a subset of patients, MP may represent the expression of a mesenteric fibroinflammatory response occurring in the context of chronic immune activation associated with IBD [7,10,28,29,43,44,45,46,47]. This interpretation, however, remains speculative in the absence of dedicated mechanistic studies specifically addressing the MP–IBD relationship.

**Figure 3:**
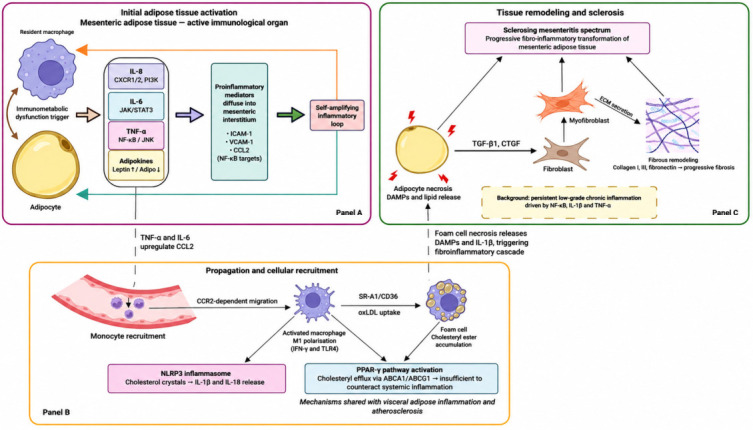
Immunometabolic pathogenesis of mesenteric panniculitis. (**Panel A**)—Initial activation of mesenteric adipose tissue as an active immunological organ. Resident macrophages and adipocytes respond to immunometabolic triggers by releasing pro-inflammatory cytokines (IL-6/JAK-STAT3, TNF-α/NF-κB/JNK, IL-8/CXCR1/2-PI3K) and adipokines (leptin ↑, adiponectin ↓), promoting inflammatory mediator release and CCL2-dependent monocyte recruitment. (**Panel B**)—Propagation of inflammation through CCR2-dependent monocyte migration, M1 macrophage polarization, oxLDL uptake, foam cell formation, NLRP3 inflammasome activation, and IL-1β/IL-18 release. PPAR-γ-mediated cholesterol efflux via ABCA1/ABCG1 is insufficient to counterbalance the inflammatory response. (**Panel C**)—Progressive fibroinflammatory remodeling. Adipocyte and foam cell necrosis release DAMPs and IL-1β, activating the TGF-β1/CTGF pathway, fibroblast-to-myofibroblast transition, extracellular matrix secretion, and collagen I/III and fibronectin deposition, leading to fibrosis within the sclerosing mesenteritis spectrum. Background: persistent low-grade chronic inflammation driven by NF-κB, IL-1β, and TNF-α sustains all three phases.

The pathophysiological mechanisms are illustrated in Figure 3.

The main etiological factors and pathogenic mechanisms implicated in MP are summarized in Table 2.

### 3.3. Diagnosis of Mesenteric Panniculitis

#### 3.3.1. Positive Diagnosis

The clinical diagnosis of MP is challenging, as the presentation is non-specific and heterogeneous. Patients may be asymptomatic or may present with vague abdominal symptoms, most commonly abdominal pain without distinctive features, occasionally associated with bowel habit disturbances, nausea, or weight loss. Systemic manifestations, such as fever or asthenia, may be present but are inconsistent. Physical examination is often normal; however, in some cases, it may reveal nonspecific abdominal tenderness or, less frequently, a palpable mass [13,18,52].

Laboratory investigations are non-specific, potentially revealing an inflammatory syndrome of variable intensity or values within normal limits, which limits the diagnostic value of clinical assessment alone [52].

In this context, the diagnosis of MP is predominantly imaging-based, with contrast-enhanced multidetector CT representing the method of choice. In the majority of cases, the diagnosis can be established on the basis of a typical imaging pattern, without the need for histological confirmation, in the absence of clinical or imaging red flags for malignancy [11,14,29].

The characteristic CT appearance consists of a hyperattenuating mesenteric fat area, typically located at the level of the small bowel mesenteric root, frequently involving the jejunal mesentery, associated with displacement of bowel loops and encasement of vascular structures without direct invasion. Mesenteric lymphadenopathy is common and, in the majority of cases, sub-centimeter in size [7,11,53].

Suggestive imaging findings include hyperattenuation of the mesenteric fat, small mesenteric nodules, the “fat ring” sign (preservation of perivascular fat), and a peripheral pseudocapsule. The combination of these features supports the benign inflammatory nature of the condition and contributes to differentiation from neoplastic infiltrations [8,54,55].

The imaging criteria proposed by Coulier are widely used and define the diagnosis when at least three of the following are present: a well-defined mesenteric fat mass, increased fat attenuation, mesenteric nodules, the “fat ring” sign, and a pseudocapsule [19,56].

No specific serological markers exist, and biopsy is not routinely required in typical presentations. Histological confirmation is reserved for cases with atypical imaging appearance or suspicion of malignancy [11,14].

#### 3.3.2. Differential Diagnosis

The differential diagnosis of MP is broad and includes neoplastic, inflammatory, infectious, and vascular processes. Differentiation relies on the correlation of clinical and imaging findings and on the identification of red flags, such as bulky lymphadenopathy, increased fluorodeoxyglucose (FDG) uptake on positron emission tomography-computed tomography (PET-CT), or invasion of adjacent structures (Table 3) [13,52].

### 3.4. Treatment of Mesenteric Panniculitis

The management of MP is individualized based on symptom presence and severity, clinical impact, and the occurrence of complications, in the absence of standardized therapeutic recommendations. In the majority of asymptomatic cases, a conservative approach is preferred, given the frequently benign and stable nature of the disease, while pharmacological treatment is reserved primarily for symptomatic forms. Available data support a symptom-guided strategy, in which the primary objective is clinical improvement rather than imaging normalization, as radiological changes may persist despite a favorable symptomatic response [13,19,44].

Therapeutic options reported in the literature include clinical observation, systemic corticosteroid therapy, antifibrotic and anti-inflammatory therapies, and, in selected cases, immunosuppressive agents, immunomodulatory therapies, or surgical treatment. The main indications and characteristics of these approaches are summarized in Table 4 [9,29,60,61].

## 4. Association of MP with IBD

### 4.1. Epidemiology

Epidemiological data on the association between MP and IBD remain limited and heterogeneous. In CT-based series, IBD is reported as a relatively rare comorbidity, frequently without differentiation between CD and UC and without systematic correlation with disease activity or the temporal relationship to the diagnosis of MP, precluding precise estimation of the true frequency or specificity of the association [20,21,26].

Of the two major forms of IBD, the association appears better supported for CD than for UC. In the retrospective analysis by Protin-Catteau et al., CD was identified in 4.2% of patients with MP, suggesting a non-random overlap between mesenteric inflammation and chronic intestinal inflammatory disease [44].

Additional data derived from clinical observations and small series reporting CD among the autoimmune or inflammatory comorbidities associated with MP, supporting the existence of a common immunological background, albeit without demonstrating a direct causal relationship [45,64].

In contrast, for UC, data remain fragmentary, limited to isolated observations or mentions within heterogeneous cohorts. In the cohort analyzed by Küpeli et al. [43], UC was reported among the most common accompanying benign disorders in 3 out of 309 MP patients (0.97%), without dedicated analysis of this association. The narrative review by Ayala Gutiérrez and de Ramón Garrido [21], which synthesized data from 202 published articles encompassing 1305 patients with MP, identified UC as a personal history in 1 of 710 patients (0.14%). Additionally, IBD was considered a possible etiological factor in 7 out of 1243 patients (0.6%), without differentiation between CD and UC. Collectively, these data suggest a possible but rare and insufficiently characterized association between MP and UC [21,43].

Overall, the available literature suggests that the association between MP and IBD is likely underrecognized and insufficiently defined epidemiologically. To date, the relationship appears more plausible and better supported for CD, whereas for UC, the evidence remains limited to isolated reports and small retrospective series [28,29].

Studies reporting the association between MP and IBD are summarized in Table 5.

### 4.2. Common Pathogenic Mechanisms

Interest in the association between MP and IBD stems from recognition of the mesentery as an active immunological compartment directly involved in perpetuating inflammation and fibrotic remodeling. In CD, inflammation is not confined to the intestinal mucosa but may become transmural and extramural, involving adjacent mesenteric lymphatic, vascular, and adipose structures, thereby providing a plausible biological framework for interpreting MP as an expression of the same persistent inflammatory activation [65,66].

In CD, imaging and experimental data indicate that mesenteric fat hypertrophy, fibroadipose proliferation, and lymphadenopathy correlate with transmural inflammation and postoperative recurrence, supporting the concept that the mesentery may serve as an active source of inflammation and fibrogenesis [39,67,68].

Mechanistically, mesenteric adipose tissue is an active immunometabolic organ that secretes pro-inflammatory cytokines and adipokines, including TNF-α, IL-1β, IL-6, leptin, and other profibrogenic mediators. In this model, the interaction between mesenteric adipocytes, macrophages, mesenteric lymph nodes, and the gut microbiota may sustain an autonomous inflammatory microenvironment capable of perpetuating both intestinal inflammation and fibroinflammatory remodeling of the mesentery [34,69,70].

Specific profibrotic mechanisms, including activation of the autotaxin–lysophosphatidic acid axis and mesenteric lymphatic dysfunction, may further amplify fibrotic remodeling even in the absence of overt mucosal disease activity. These data support the concept of an intestino-mesenteric continuum, in which MP may represent a fibroinflammatory mesenteric phenotype potentially integrable, at least in some settings, within the spectrum of IBD, particularly CD [40,58,71].

For UC, the biological arguments linking MP to the underlying disease are less compelling than for CD. Unlike Crohn’s disease, UC is primarily characterized by inflammation confined to the mucosa and submucosa, without transmural extension, and mesenteric involvement is correspondingly limited. Accordingly, mesenteric hypertrophy, creeping fat, and extensive fibroadipose remodeling, which are characteristic features of CD, are not observed in UC, providing a biologically plausible explanation for the rarity of MP in this setting [41]. Nevertheless, UC is recognized as a systemic immune-mediated disease, with inflammatory mechanisms extending beyond the intestinal mucosa and giving rise to well-established extraintestinal immune manifestations [47]. Based on these observations, one possible interpretation is that MP occurring in a patient with UC may represent either a coincidental idiopathic fibroinflammatory process or, alternatively, an immune-mediated mesenteric response developing in the setting of systemic immune dysregulation rather than as a consequence of direct transmural intestinal inflammation. However, dedicated mechanistic studies specifically addressing MP in UC are currently lacking, and this hypothesis remains speculative pending prospective validation [11].

### 4.3. Diagnostic Pitfalls

One of the main challenges in interpreting MP in patients with IBD is the imaging overlap between primary mesenteric inflammation and secondary mesenteric changes due to intestinal disease. Increased mesenteric fat attenuation, described under the term “misty mesentery,” may occur in active CD through focal inflammation adjacent to affected intestinal segments, lymphadenopathy, and occasionally mesenteric abscesses, potentially mimicking idiopathic MP [53,55].

In certain settings, mesenteric inflammation may even precede overt intestinal manifestations. The report by Nuzzo et al. demonstrated that isolated ileal CD may simulate idiopathic MP over an extended period, in the context of repeatedly normal endoscopies and non-specific biological markers, with the diagnosis being clarified only after progression of mesenteric changes and subsequent identification of a mildly inflammatory and stricturing ileal segment at laparoscopy [72]. Of note, a case of IgG4-related sclerosing mesenteritis associated with CD was previously reported by Kim et al., representing one of the earliest documented cases of overlap between mesenteric fibroinflammatory conditions and IBD [73]. Together with the report by Nuzzo et al., these observations suggest that the association between mesenteric fibroinflammatory conditions and CD, while biologically plausible, remains exceptionally rare and insufficiently characterized in the literature. This observation underscores the necessity of systematically ruling out small bowel involvement before labeling MP as idiopathic in patients with suspected IBD [72].

Another important source of diagnostic error is represented by fibroinflammatory or neoplastic processes that may reproduce the same imaging pattern. IgG4-related sclerosing mesenteritis, lymphoma, and other mesenteric infiltrations may mimic both MP and IBD-associated mesenteric involvement, with histological confirmation being required in atypical or pseudotumoral presentations [52,74].

Furthermore, in imaging-based cohorts, IBD is frequently reported without stratification between CD and UC, which may generate additional diagnostic confusion and limit the interpretation of the true relationship between the two entities. In this context, accurate differentiation requires careful integration of clinical, endoscopic, and imaging data, as well as the active exclusion of small bowel pathology, malignancy, and IgG4-related disease in atypical presentations [21,26].

## 5. Mesenteric Panniculitis as an Immunological Reaction/Drug-Induced Adverse Effect

Iatrogenic cases of MP have been described, particularly in the context of modern oncological therapies. Immune checkpoint inhibitors and targeted therapies may induce aberrant inflammatory reactions, including in mesenteric adipose tissue, through excessive immune activation and loss of peripheral tolerance. These forms fall within the spectrum of immune-mediated adverse reactions and may occur in isolation or in association with other systemic inflammatory manifestations [38,51].

Similarly, drug reactions are mentioned as potential triggering factors of mesenteric inflammation, although direct causal relationships remain difficult to demonstrate, and the majority of available data derive from case reports and small clinical series, in the absence of controlled studies confirming this association [30,31].

Vedolizumab is a humanized monoclonal antibody that acts through selective blockade of the α4β7 integrin, inhibiting the migration of gut-tropic T lymphocytes towards the intestinal mucosa by interfering with its interaction with MAdCAM-1, an adhesion molecule preferentially expressed on intestinal endothelium [75].

Although vedolizumab’s gut-selective mechanism of action is associated with a favorable systemic safety profile, clinical observations have documented the de novo occurrence of extraintestinal inflammatory manifestations in patients receiving this therapy [76,77]. The underlying mechanisms remain incompletely understood, and whether these events reflect a direct drug-related effect, loss of prior systemic immunosuppression following anti-TNF discontinuation, or coincidental immune phenomena cannot be determined from available data [78].

Observational clinical data have reported de novo EIMs in 26.7% of patients treated with vedolizumab in a retrospective study of 71 patients, with articular and cutaneous manifestations being the most frequently reported extraintestinal manifestations [76].

Consistently, the French multicenter GETAID OBSERV-IBD cohort, which included 294 patients followed up to week 54, reported 34 incident cases (13.8%) of arthralgias or inflammatory arthritis and 14 cases (4.8%) of paradoxical cutaneous manifestations occurring de novo under treatment [78].

However, the interpretation of these observations is complicated by important confounding factors, particularly prior exposure to anti-TNF-α therapies [77].

In the absence of previous reports explicitly documenting an association between vedolizumab and MP, the potential relationship observed in the present case should be interpreted with caution. Although the onset of mesenteric panniculitis occurred during ongoing vedolizumab therapy, complete clinical and radiological remission was achieved without treatment discontinuation, which argues against a direct drug-induced mechanism. Nevertheless, a potential contribution of vedolizumab cannot be entirely excluded. Several alternative explanations remain equally plausible: idiopathic mesenteric panniculitis, an unidentified infectious trigger, an immune-mediated process independent of current biological therapy, or the potential influence of prior pharmacological exposure. Consequently, any potential association with vedolizumab should be interpreted with caution and should not be considered indicative of a drug-induced adverse effect.

## 6. Strengths, Limitations, and Future Perspectives

To the best of our knowledge, this article describes a rare case of MP associated with UC in a patient receiving vedolizumab therapy, in the context of biological, endoscopic, and histological remission of IBD. The originality of this case lies in the dissociation between mucosal inflammatory control and the occurrence of mesenteric inflammation. The article also provides a structured synthesis of available literature data on the association between MP and IBD, offering a critical appraisal of existing studies and highlighting the low level of evidence in this field.

The limitations of this article stem from its case report design, which precludes establishing a causal relationship between MP and UC, or between MP and vedolizumab therapy.

In addition to the inherent limitations of a single case report, several case-specific diagnostic limitations must be acknowledged. The diagnosis of MP was established on the basis of characteristic contrast-enhanced computed tomography criteria, in the absence of histopathological confirmation. Following complete clinical and radiological remission after medical treatment, the mesenteric inflammatory changes had resolved, precluding subsequent histopathological confirmation. Although imaging-based diagnosis is widely accepted in patients with typical radiological features, the absence of tissue confirmation limits the definitive exclusion of alternative fibroinflammatory conditions, including IgG4-related disease and disorders within the retroperitoneal fibrosis spectrum. This limitation is particularly relevant given the novelty of the reported association and the hypothesis-generating nature of the present observation.

Although the diagnosis was supported by characteristic imaging findings and an extensive diagnostic work-up—including microbiological, immunological, endoscopic, and histological investigations, as summarized in Table 1—several additional evaluations could not be performed. Specifically, extractable nuclear antigen (ENA) antibody profiling and serum complement levels (C3 and C4) are not routinely available at our institution. Similarly, vedolizumab trough levels and anti-drug antibody testing were not available. Positron emission tomography-computed tomography (PET-CT) was not performed because this imaging modality is unavailable at our institution; however, the contrast-enhanced CT findings were consistent with established radiological criteria for MP and showed no features suggestive of occult malignancy.

The interpretation of the available literature is further limited by the predominance of retrospective studies, small case series, heterogeneous imaging criteria, inconsistent terminology, and the lack of systematic histopathological confirmation, all of which reduce diagnostic standardization and comparability across studies [11,19]. Selection bias introduced through retrospective radiological searches, the non-uniform use of biopsy, and the absence of standardized follow-up protocols additionally hamper the differentiation of clinically relevant forms from incidental findings and limit clarification of the prognostic significance of this entity [26,27]. Current therapeutic recommendations remain largely empirical, based on limited clinical experience rather than randomized controlled trials [14,61], while the reported associations of MP with both malignancy and IBD remain insufficiently characterized because of methodological heterogeneity and the absence of dedicated prospective studies, particularly for UC [10,47,79]. Furthermore, it remains unclear whether mesenteric adipose tissue remodeling represents a cause or a consequence of intestinal inflammation, limiting the precise definition of the mesentery as a potential therapeutic target [41,71].

Taken together, these case-specific and literature-related limitations indicate that the observed association should be interpreted with caution. In the absence of histopathological confirmation and given the inherent limitations of a single-case report, no causal relationship between MP, UC, and vedolizumab therapy can be established. Furthermore, the delayed onset of MP—approximately three years after vedolizumab initiation and four weeks after the last infusion—constitutes an additional argument against a direct pharmacological causal relationship with vedolizumab.

Future prospective multicenter studies integrating clinical, imaging, histopathological, immunological, and molecular data are needed to standardize diagnostic criteria, clarify the relationship between mesenteric and intestinal inflammation in IBD, and better define the clinical significance of mesenteric involvement. Such studies may also help determine whether MP represents an IBD-associated mesenteric fibroinflammatory phenomenon or a distinct fibroinflammatory disorder.

## 7. Conclusions

MP is a fibroinflammatory condition of the mesentery characterized by variable clinical expression and predominantly imaging-based diagnosis, with its clinical significance incompletely defined. Available data suggest that MP and IBD may share common immunological and fibroinflammatory pathways. However, current clinical evidence supporting this association is substantially stronger for CD than for UC, in which published evidence remains limited.

The concept of the intestine–mesentery continuum does not exclude the possibility that inflammatory processes may predominantly involve the mesentery while intestinal disease remains clinically quiescent. This observation supports the need to evaluate mesenteric pathology independently of intestinal disease activity in selected clinical scenarios.

Although immune-mediated reactions, including paradoxical manifestations, have been reported in association with biological therapies, establishing a causal relationship between MP and vedolizumab—or any biological agent—remains challenging in the absence of controlled data and dedicated mechanistic studies.

In this context, clarification of these associations requires dedicated prospective studies systematically integrating clinical, imaging, histopathological, and biological data, including in the context of gut-selective biological therapies.

The present case, describing the co-occurrence of MP and UC in a patient receiving vedolizumab during sustained clinical, endoscopic, and histological remission, contributes to the limited available evidence regarding this association. Although it does not establish a causal relationship between MP, UC, or vedolizumab therapy, it supports further prospective investigation of the mesenteric compartment in IBD while underscoring the hypothesis-generating nature of this single observation.

## Figures and Tables

**Table 1:** Laboratory investigations, infectious workup, autoimmune serological screening, and endoscopic assessment at the time of presentation.

Investigation	Result
**Laboratory investigations**	
Leukocyte count	14,258/mm^3^ (reference: 4000–10,000/mm^3^)
C-reactive protein (CRP)	5.23 mg/dL (reference: <0.5 mg/dL)
Fecal calprotectin	20.3 µg/g (reference: <50 µg/g)
Liver function tests (AST, ALT, ALP, GGT, bilirubin)	Within normal limits
Renal function tests (serum creatinine, urea)	Within normal limits
Serum electrolytes	Within normal limits
**Infectious workup**	
Clostridioides difficile: GDH, toxins A and B	Negative
Stool culture	Negative
Stool parasitology	Negative
Urine culture	Negative
Blood cultures	Negative
Chest X-ray	No pathological findings
**Autoimmune and immunological investigations**	
IgG4	0.5 g/L (reference: 0.03–2.01 g/L)
Antinuclear antibodies (ANA)	Negative (index <0.7; reference: <0.7)
Anti-neutrophil cytoplasmic antibodies (ANCA)	Negative (titre <1:10; reference: <1:10)
Rheumatoid factor (RF)	2 IU/mL (reference: <14 IU/mL)
**Assessment of ulcerative colitis activity**	
Endoscopic Mayo Score	0 (endoscopic remission)
Nancy Histological Index	0 (histological remission)

Abbreviations: AST, aspartate aminotransferase; ALT, alanine aminotransferase; ALP, alkaline phosphatase; GGT, gamma-glutamyl transferase; IgG4, immunoglobulin G subclass 4; GDH, glutamate dehydrogenase.

**Table 2:** Etiology and associated factors in mesenteric panniculitis (MP).

Etiological Category	Representative Examples	Proposed Mechanism	References
Mechanical/ postoperative factors	Abdominal surgery; abdominal trauma	Aberrant tissue repair	[8,20]
Vascular factors	Mesenteric ischemia, vasculitis	Secondary inflammatory activation	[30]
Infectious factors	Chronic gastrointestinal infections, (including *Helicobacter pylori*)	Persistent immune activation	[34,48]
Autoimmune/ fibroinflammatory factors	Retroperitoneal fibrosis, sclerosing cholangitis, Riedel’s thyroiditis	Shared immunological substrate	[8,20]
IgG4-related disease	Forms of IgG4-associated sclerosing mesenteritis	Immune-mediated fibroinflammation	[14,49]
Neoplastic factors	Lymphomas, solid neoplasms (e.g., prostate)	paraneoplastic phenomenon or detection bias	[19,22,50]
Metabolic factors	Visceral obesity; metabolic syndrome; MASLD	metabolic inflammation	[35,36,37]
Immunometabolic factors	Secretion of TNF-α, IL-6, IL-8, leptin, MCP-1; macrophage activation	mesenteric fibrotic remodeling	[32]
Drug-induced/ iatrogenic factors	Oncological therapies, immune checkpoint inhibitors, targeted therapies	Drug-induced immune reactions	[14,38,51]
Intestinal inflammatory factors	CD, UC	intestinal-mesenteric immune activation	[39,40,41,42]
Idiopathic factors	Cases without identifiable cause	Unknown mechanism	[30]

**Table 3:** Differential diagnosis of MP.

Entity	Suggestive Features	Differentiating Features	References
Mesenteric lymphoma	Bulky confluent lymphadenopathy	Enlarged (>10–12 mm) FDG-avid lymph nodes	[53,57]
Peritoneal carcinomatosis	Peritoneal nodules, ascites	Peritoneal dissemination, metastases	[30]
Neuroendocrine tumors (carcinoid)	Mesenteric mass with desmoplastic reaction	Calcifications; retractile fibrosis	[30]
Desmoid tumors	Infiltrative solid mass	Absence “fat ring” sign; progressive growth	[30]
Liposarcoma	Bulky adipose mass	Solid component, heterogeneity	[30]
IgG4-related disease	Fibroinflammatory mass	Multiorgan involvement, elevated serum IgG4	[49]
Peritoneal tuberculosis	Lymphadenopathy, ascites	Evidence of systemic infection	[52]
CD (mesenteric)	“Creeping fat”; bowel wall thickening	Active intestinal lesions	[58]
Mesenteric edema	Diffuse fat stranding	Underlying Cardiac/hepatic disease; reversibility	[59]
Mesenteric venous thrombosis	Edema, congestion	Evident vascular thrombosis	[59]

**Table 4:** Therapeutic options in MP.

Therapeutic option	Examples	Indications	Efficacy/Remarks	References
Watchful waiting	Clinical monitoring	Asymptomatic patients	Often stable or self-limited	[13,14]
Corticosteroid therapy	Prednisone	Symptomatic forms	First-line treatment; frequent clinical response	[13,14]
Antifibrotic therapy	Tamoxifen	Combined with corticosteroid therapy	Reported benefit in combination therapy	[13,14]
Anti-inflammatory therapy	Colchicine	Alternative/ adjuvant	Reported benefit in selected series	[29,60,61]
Immunosuppressive agents	Azathioprine, methotrexate	Refractory forms	Limited evidence	[29,60,61]
Other therapies	Pentoxifylline, thalidomide, biologics	Selected cases	Limited evidence	[29,60,61]
Symptomatic treatment	NSAIDs, antibiotic therapy	Mild forms	Possibly self-limiting course	[62,63]
Surgical treatment	—	Complications	Limited indications	[9,13]

Abbreviations: NSAIDs = non-steroidal anti-inflammatory drugs.

**Table 5:** Studies reporting the association between MP and IBD.

Study	Study Design	Patients (*n*)	MP Cases	Key Findings
Smith et al., 2013 [26]	Retrospective CT-based cohort	359	359	IBD reported as a rare comorbidity in 6/359 patients (1.6%), without differentiation between CD and UC
Protin-Catteau et al., 2016 [44]	Retrospective CT-based matched-pair analysis	3054	96	CD was identified in 4.2% of patients with MP
Ayala Gutiérrez & de Ramón Garrido, 2016 [21]	Narrative review of 202 published articles	1305	1305	UC identified as a personal history in 1/710 patients (0.14%); IBD considered a possible etiological factor in 7/1243 patients (0.6%), CD and UC were not differentiated
Nyberg et al., 2017 [64]	Retrospective clinical and imaging cohort	31	27	CD reported in 1/27 MP patients (3.7%) among autoimmune comorbidities; no dedicated statistical analysis was performed
Küpeli et al., 2018 [43]	Retrospective CT-based cohort	22,033	309	UC reported among benign comorbidities in 3/309 patients (0.97%), without a dedicated analysis of the association

## Data Availability

The original contributions presented in the study are included in the article; further inquiries can be directed to the corresponding author.

## Abbreviations

The following abbreviations are used in this manuscript:

MP	Mesenteric panniculitis
IBD	Inflammatory bowel disease
UC	Ulcerative colitis
CD	Crohn’s disease
EIM	Extraintestinal manifestation
CT	Computed tomography
MRI	Magnetic resonance imaging
CRP	C-reactive protein
FDG	Fluorodeoxyglucose
PET-CT	Positron emission tomography-computed tomography
PET	Positron emission tomography
MASLD	Metabolic dysfunction-associated steatotic liver disease
NAFLD	Non-alcoholic fatty liver disease
NSAIDs	Non-steroidal anti-inflammatory drugs
PPAR-γ	Peroxisome proliferator-activated receptor gamma
MAdCAM-1	Mucosal addressin cell adhesion molecule-1
ICI	Immune checkpoint inhibitors
ATX-LPA	Autotaxin–lysophosphatidic acid axis
TGF-β1	Transforming growth factor beta-1
CTGF	Connective tissue growth factor
NF-κB	Nuclear factor kappa B
IL-1β	Interleukin-1 beta
IL-6	Interleukin-6
IL-8	Interleukin-8
IL-18	Interleukin-18
TNF-α	Tumor necrosis factor alpha
CCL2	C-C motif chemokine ligand 2
NLRP3	NLR family pyrin domain containing 3
DAMPs	Damage-associated molecular patterns
ECM	Extracellular matrix
MDCT	Multidetector computed tomography
ICAM-1	Intercellular adhesion molecule-1
VCAM-1	Vascular cell adhesion molecule-1
COX-2	Cyclooxygenase-2
JAK/STAT3	Janus kinase/signal transducer and activator of transcription 3
ABCA1/ABCG1	ATP-binding cassette transporter A1/G1
SR-A1/CD36	Scavenger receptor A1/CD36
ANA	Antinuclear antibodies
ANCA	Anti-neutrophil cytoplasmic antibodies
RF	Rheumatoid factor
ENA	Extractable nuclear antigen
IgG4	Immunoglobulin G subclass 4
GDH	Glutamate dehydrogenase
CXCR1/2	C-X-C motif chemokine receptor 1/2
PI3K	Phosphoinositide 3-kinase
JNK	c-Jun N-terminal kinase
oxLDL	Oxidized low-density lipoprotein
IFN-γ	Interferon gamma
TLR4	Toll-like receptor 4
